# A new continuous noninvasive finger cuff device (Vitalstream) for cardiac output that communicates wirelessly via bluetooth or Wi-Fi

**DOI:** 10.1186/s12871-023-02114-z

**Published:** 2023-05-25

**Authors:** Irwin Gratz, Martin Baruch, Ahmed Awad, Brian McEniry, Isabel Allen, Julia Seaman

**Affiliations:** 1 Cooper University Hospital, Camden, NJ USA; 2 Caretaker Medical, Charlottesville, VA USA; 3 University of California, San Francisco, CA USA; 4 Bayview Analytics, Oakland, CA USA

**Keywords:** Cardiac output, Non-invasive, Thermodilution, Finger cuff, Cardiac surgery, Agreement, Wi-Fi Wireless device

## Abstract

**Background:**

The new noninvasive Vitalstream (VS) continuous physiological monitor (Caretaker Medical LLC, Charlottesville, Virginia), allows continuous cardiac output by a low pump-inflated, finger cuff that pneumatically couples arterial pulsations via a pressure line to a pressure sensor for detection and analysis. Physiological data are communicated wirelessly to a tablet-based user interface via Bluetooth or Wi-Fi. We evaluated its performance against thermodilution cardiac output in patients undergoing cardiac surgery.

**Methods:**

We compared the agreement between thermodilution cardiac output to that obtained by the continuous noninvasive system during cardiac surgery pre and post-cardiac bypass. Thermodilution cardiac output was performed routinely when clinically indicated by an iced saline cold injectate system. All comparisons between VS and TD/CCO data were post-processed. In order to match the VS CO readings to the averaged discrete TD bolus data, the averaged CO readings of the ten seconds of VS CO data points prior to a sequence of TD bolus injections was matched. Time alignment was based on the medical record time and the VS time-stamped data points. The accuracy against reference TD measurements was assessed via Bland–Altman analysis of the CO values and standard concordance analysis of the ΔCO values (with a 15% exclusion zone).

**Results:**

Analysis of the data compared the accuracy of the matched measurement pairs of VS and TD/CCO VS absolute CO values with and without initial calibration to the discrete TD CO values, as well as the trending ability, i.e., ΔCO values of the VS physiological monitor compared to those of the reference. The results were comparable with other non-invasive as well as invasive technologies and Bland-Altman analyses showed high agreement between devices in a diverse patient population. The results are significant regarding the goal of expanding access to effective, wireless and readily implemented fluid management monitoring tools to hospital sections previously not covered because of the limitations of traditional technologies.

**Conclusion:**

This study demonstrated that the agreement between the VS CO and TD CO was clinically acceptable with a percent error (PE) of 34.5 to 38% with and without external calibration. The threshold for an acceptable agreement between the VS and TD was considered to be below 40% which is below the threshold recommended by others.

## Introduction

Fluid management is a critical aspect of patient care; however, monitoring technologies for assessing fluid status or the need for administration of fluids have traditionally been restricted to the care settings in the OR and ICUs. The need for effective fluid management tools is significant in other settings. One example is the ER, where invasive technologies such as arterial and Swan-Ganz catheters are rarely found, yet the need for assessing and adjusting fluid status is often critical. A number of non-invasive devices have attempted to fill this void, but introduction has been limited due to device size, technical complexities, patient comfort, cost issues, and accuracy [1, 2]. There remains a need to measure cardiac output (CO) noninvasively by a clinically validated technique; ideally, by means of wireless and small-footprint technologies that can be utilized quickly and effectively in many environments.

The Vitalstream (VS) continuous noninvasive physiological monitor (Caretaker Medical LLC, Charlottesville, Virginia, further referred to as CTM) is FDA-cleared for the measurement of heart rate, continuous noninvasive BP, and respiratory rate as well as advanced hemodynamic parameters of cardiac output/stroke volume, left ventricular ejection time and heart rate variability. The system and the underlying approach have been described in detail elsewhere [3, 4]. Briefly, the VS tracks central aortic BP via pulse analysis, specifically Pulse Decomposition Analysis (PDA), of the peripheral pulse at a distal site, typically the finger. The device uses a low pressure [30–40 mmHg], pump-inflated, finger cuff that pneumatically couples arterial pulsations via a pressure line to a pressure sensor for detection and analysis. Physiological data are communicated wirelessly to a tablet-based user interface via Bluetooth or Wi-Fi. The device is currently cleared only for adult use, i.e., patients 18 years and older. There are no regulatory restrictions on the clinical use of the device, however, just like all finger-based sensing technologies, extremely cold fingers can impede operation.

The PDA approach is based on the concept that primarily two central reflection sites are responsible for the shape of the pressure pulse envelope of the upper body [5–7]. The two reflection sites, one located at the aortic juncture of thoracic and abdominal aortas, and the other at the iliac bifurcation, reflect the primary left ventricular ejection pulse to give rise to two additional, reflected, component pulses that trail the primary ejection pulse, as a result of which, within the pulse pressure envelope of each cardiac cycle, these three component pulses arrive sequentially in the arterial periphery. The model of the spatio-temporal behavior of these three component pulses constitutes the PDA formalism that can be used to monitor hemodynamic states and changes. The PDA model is based on physical assumptions that are readily testable and which coherently explain the structure of the pulse. The purpose of this study was to validate cardiac output readings, provided by the Vitalstream, both in magnitude and trend direction, against Gold Standard measurements obtained using thermodilution (TD) as well as to assess relative time response characteristics.

CTM’s approach to calculating cardiac output (CO) utilizes a linear model that incorporates arterial stiffness estimation, impedance correction, and integration over the “systolic” area of the pressure pulse [8]. A problem with traditional pulse contour approaches has been the estimation of the systolic area, since these approaches frequently simply utilize the “dichrotic” notch to separate systolic and diastolic phases. However, this categorization yields integration over both the actual systolic component pulse area as well as sections of reflected component pulses that complete the pulse envelope, yielding inaccurate systolic area estimates. PDA, which offers a comprehensive and physical explanation of the arterial pulse envelope morphology, provides an opportunity to refine the systolic area calculation.

Setting realistic a-priori expectations, particular in the context of evaluating a non-invasive technology is important as the measurement of cardiac output is a somewhat approximate science. Not only do invasive approaches routinely have performance errors well in excess of 40%, but recent comparisons between Gold Standards have demonstrated similar discrepancies [9]. We refer here to the Fick/TD comparison results reported in Fares, who reported a standard deviation of 2.03 l/min, corresponding to an estimated error larger than 65% [10]. Large discrepancies were also reported by Opotowsky and Tehrani [11, 12]. With these considerations in mind acceptable agreements were errors less than 40% and concordance higher than 0.8.

Such errors can correspond to LOAs on the order of 2.5 l/min, which is highly significant even at an average CO of 5 to 6 l/min. However, this has been the effective state of clinical practice, just one example being the frequently required additional TD boluses to lower the variance of a shot sequence to an acceptable limit. For clinicians needing to make treatment decisions, the potential added benefits with an equivalently accurate but continuous and non-invasive technology like the Vitalstream is the enhanced ability to identify trends and to assess the variability.

In what follows we present the results of comparing the VS cardiac output absolute and trending measurements with those obtained from discrete bolus thermodilution (TD) as well as from continuous cardiac output monitoring (CCO) in patients undergoing cardiac surgery.

### Methods

At Cooper University Hospital patients older than 18y undergoing cardiac surgery in whom pulmonary artery catheter placement was planned as part of their care, were recruited from April 2021 to January 2022 in this IRB approved study. All patients provided written informed consent.

Continuous pulmonary artery catheters (Model #777F8, Edwards Lifesciences Cor., Irvine, CA) were inserted after anesthesia induction. Bolus TD applications were administered depending on clinical indication. For each TD measurement typically three injections were applied within 3–4 min using iced saline via a cold injectate system (Model #93,600, Edwards Lifesciences Cor., Irvine, CA) and averaged. If differences between CO measures exceeded 1 l/min, additional injections were applied. In addition, intermittent semi-real-time cardiac output measurements were obtained using the continuous pulmonary catheter-based cardiac output monitoring system (CCO). The CCO utilizes a heating element placed in the right ventricle that is triggered in a random series of heating bursts, and a thermistor placed in the pulmonary artery for detection of the heat bursts. Since the cross-correlation algorithm that establishes detection requires lengthy input sequences, the system reports changes in cardiac output with delays on the order of 5 min, as reported by others [13]. All TD were performed on the HemoSphere® advanced monitoring platform (Edwards Lifesciences Cor., Irvine, CA). In contrast, the VS’s CO response is significantly faster and primarily driven by the averaging over 30 heartbeats.

The arterial pressure pulse signal was continuously measured noninvasively using the VS device. In order not to interfere with the surgical procedures the device was placed on the patient’s wrist during the procedure setup, with the finger cuff coupled to the middle phalanx of the middle finger, and data transmission was verified, after which time no further physical interaction with the device was possible.

Operation of the device would commence after an initial blood pressure self-calibration procedure, lasting approximately 25 s, during which time the device scans the finger cuff’s coupling pressure from 0 to 250 mmHg while collecting the pressure-modulated arterial pressure pulse signal. At the end of the pressure scan, systolic and diastolic blood pressures are calculated from the processed signal envelope. Thereafter, the device was programmed to perform self-calibration scans at 5-minute intervals, operating in between in the continuous tracking mode with the finger cuff pressure collecting pulse data at a fixed baseline cuff pressure of between 20 and 45 mmHg. The coupling pressure for continuous operation is determined as part of the self-calibration procedure and held constant until the next procedure. Collected data were sent via Wi- Fi interface to an Android tablet for storage.

#### Data inclusion

With regard to the discrete TD and the CCO data, all data deemed acceptable by the attending clinicians were used in the analysis. In the case of the VS data, a custom signal/noise factor (SNF) was used to identify poor quality data sections which were excluded. The factor is based on the standard ratio of the variances of the physiological signal band to the noise band and obtained using Fourier spectral analysis over an 8-s window with 1 s overlap. The frequency range of the band associated with the physiological signal was set to 1– 10 Hz, based on data by the authors and results by others while the noise band was set to the 100–250 Hz frequency range, which is subject to ambient noise but contains no signal relevant to the base band phenomena of the arterial pressure pulse or its propagation characteristics [14]. Data sections with an SNF below 80 were excluded from the analysis.

### Statistical analysis

All comparisons between VS and TD/CCO data were post-processed. In order to match the VS CO readings to the averaged discrete TD bolus data, the averaged CO readings of the ten seconds of VS CO data points prior to a sequence of TD bolus shots was matched. Time alignment was based on the medical record time and the VS time-stamped data points. Likewise, for VS/CCO comparisons ten seconds of VS CO data were averaged bracketing the time stamp of a CCO reading.

A statistical analysis of the data compared the accuracy of the VS absolute CO values with and without initial calibration to the discrete TD CO values, as well as the trending ability, i.e., ΔCO values of the VS physiological monitor compared to those of the reference, specifically using the four-quadrant analysis described by Saugel [15]. The analysis was performed using the MATLAB software package (Natick, USA) and Stata 17.1 (StataCorp, College Station, TX).

The accuracy against reference TD measurements was assessed via correlation analysis as well as via Bland–Altman and difference count distribution analysis of the CO values and standard concordance analysis of the ΔCO values (with a 15% exclusion zone). The Bland–Altman analysis took repeated measurements per subject into account [16]. Separate correlation and Bland-Altman analyses, for calibrated and uncalibrated cases, were also performed on the high and low CO ranges, specifically below 5 l/min and above 8 l/min. A post-hoc Bland-Altman power analysis using the resulting means and standard deviations showed power to be 0.82 for all patients and 0.78 for the 39 patients with complete data.

#### CCO time delay analysis

In order to access the VS delay in measuring CO with acute changes, an iterative concordance analysis with the “real-time” data provided by the previously described CCO system used at Cooper University Hospital was performed. We follow here the example provided by Saugel, who discusses a method for assessing delays between two methodologies that measure cardiac output [15]. The approach is based on introducing delays between both methodologies’ data and observing concordance results for improvements in trending. The approach of this analysis was facilitated by calculating the cross-correlation spectrum to determine the optimum delay between both cardiac output data series.

## Results

A total of 45 patients were enrolled. Complete sets of data for 39 patients were available (m/f: 25/14, mean age: 65.7 y (SD: 8.05 y), mean BMI: 29.8 (6.21)). Patient characteristics are compiled in Table 1. In two cases the physician was unable to place the pulmonary catheter, in two cases the VS device was accidently turned off, and in two cases temporally overlapping data were not available. A total of 80 measurement pairs were analyzed for the discrete TD comparison. For the CCO comparison of continuous data a total of 2227 matched data points were obtained from 25 patients of the overall set of 39 patients.

**Table 1:** Baseline patient characteristics

	39 patients
Characteristic	Mean (SD) or N (%)
Age – mean (std) – yr.	65.7 (8.1)
Body Mass Index – mean (std. dev.) – kg/m^2^	29.8 (6.2)
Male Sex – no. (%)	27 (69.2%)
Tobacco Use – no. (%)	17 (43%)
former	16 (41%)
current	5(12.8%)
Hypertension – no. (%)	25 (64%)
Diabetes Mellitus – no. (%)	7 (17.9%)
**Indications (%)**	
Acute Coronary Syndrome, Stable Angina, or known Coronary Artery Disease	
Valvular Disease	31 (84)
Other Indications	
CABG	4 (10.3%)
CABG + VALVE	10 (25.6%)
MITRAL VALVE	12 (30.8%)
AORTIC VALVE	12 (30.8%)
MULTI VALVE	1 (2.6%)

### TD / VS comparison

**Fig. 1:**
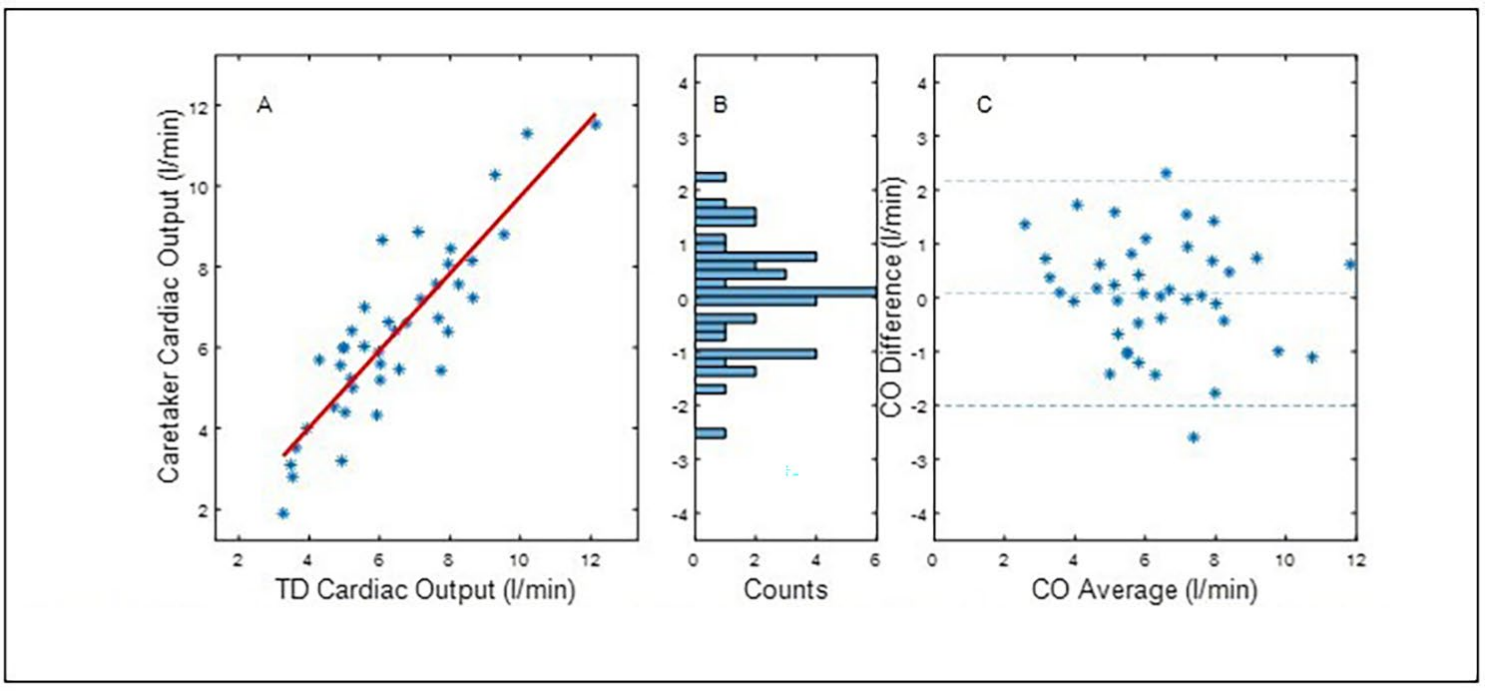
presents the statistical comparison results, correlation and Bland-Altman, for calibrating the VS CO data to the average of the first TD bolus sequence, specifically the ten seconds of data preceding the start of the sequence. The calibration data points were excluded from the graph and the analysis. Pearson Correlation (panel **A**): 0.84. Panel **B**: bin width: 0.175 l/min. Panel **C**: mean difference (accuracy): 0.08 l/min, standard deviation (precision): 1.22 l/min. Error = 34.5%. LOA: (-2.31, 2.47). 95%CI: (-0.29, 0.45)

**Fig. 2:**
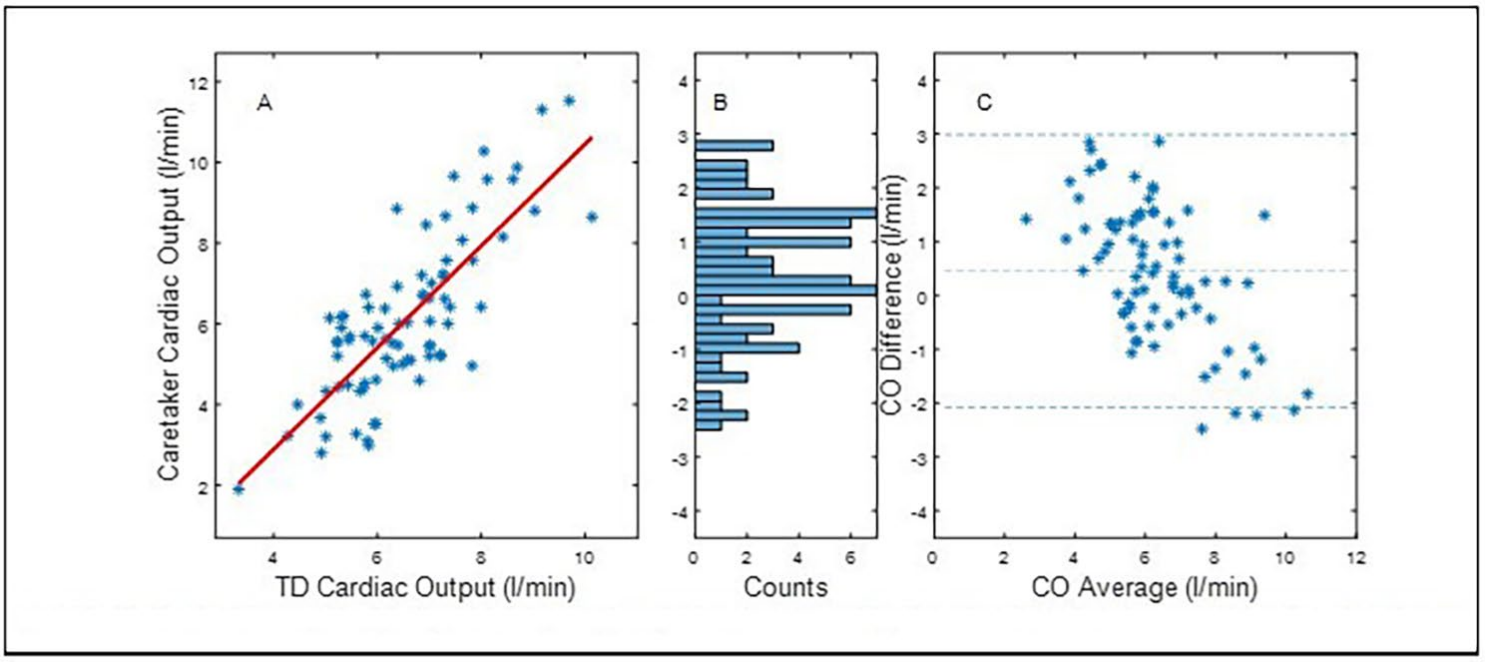
presents the corresponding statistical results without initial calibration. Pearson correlation (panel **A**): 0.79, Panel **B**: bin width: 0.175 l/min. Panel **C**: mean difference (accuracy): 0.44 l/min, standard deviation (precision): 1.27 l/min. Error = 38%. LOA: (-2.05, 2.93). 95%CI: (0.16, 0.72)

**Fig. 3:**
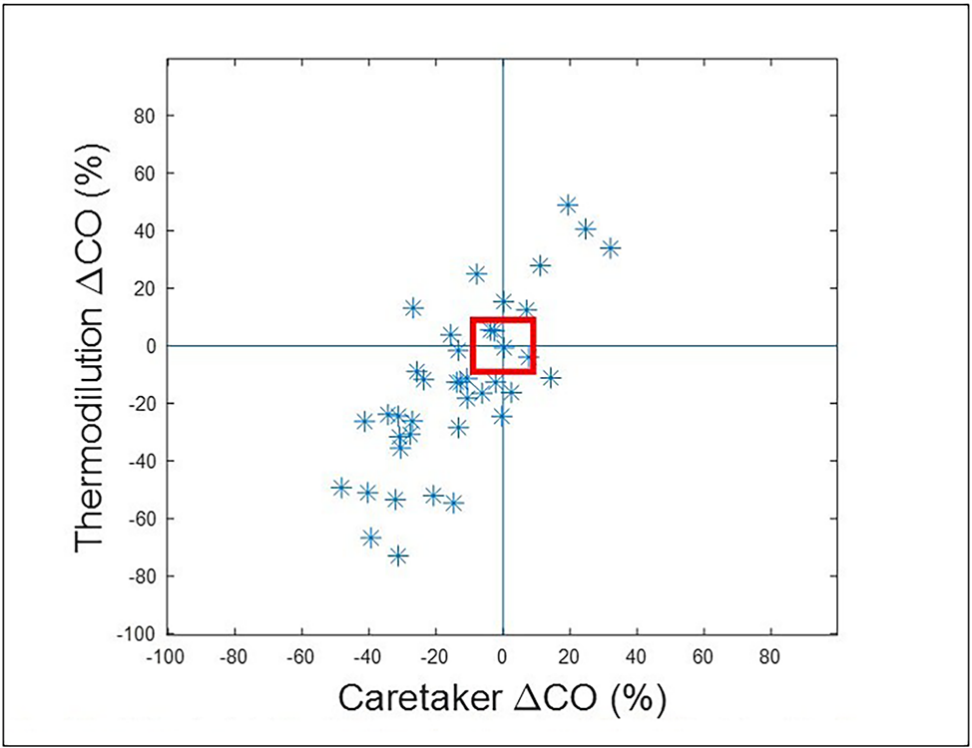
displays the results of a trend analysis. With a 15% exclusion zone, concordance is 89%. Mean difference was 0.9% (standard deviation: 18.3%), 95%CI: (82%, 90%) Results for the high and low CO ranges were as follows. For CO < 5 l/min and using initial calibration: mean difference: 0.77 l/min, standard deviation: 0.7 l/min. Error = 39%. LOA: (-0.60, 2.14). 95%CI: (0.3, 1.22). For CO < 5 l/min without initial calibration: mean difference: 1.63 l/min, standard deviation: 0.73 l/min. Error = 37%. LOA: (0.19, 3.06). 95%CI: (1.32, 1.94). For CO > 8 l/min and with initial calibration: mean difference: -0.60 l/min, standard deviation: 1.19 l/min. Error = 25%. LOA: (-2.93, 1.73). 95%CI: (-1.37, 0.18). For CO > 8 l/min without initial calibration: mean difference: -1.09 l/min, standard deviation: 1.10 l/min. Error = 23%. LOA: (-3.25, 1.07). 95%CI: (-1.65, -0.53)

#### CCO / VS comparison

**Figs. 4:**
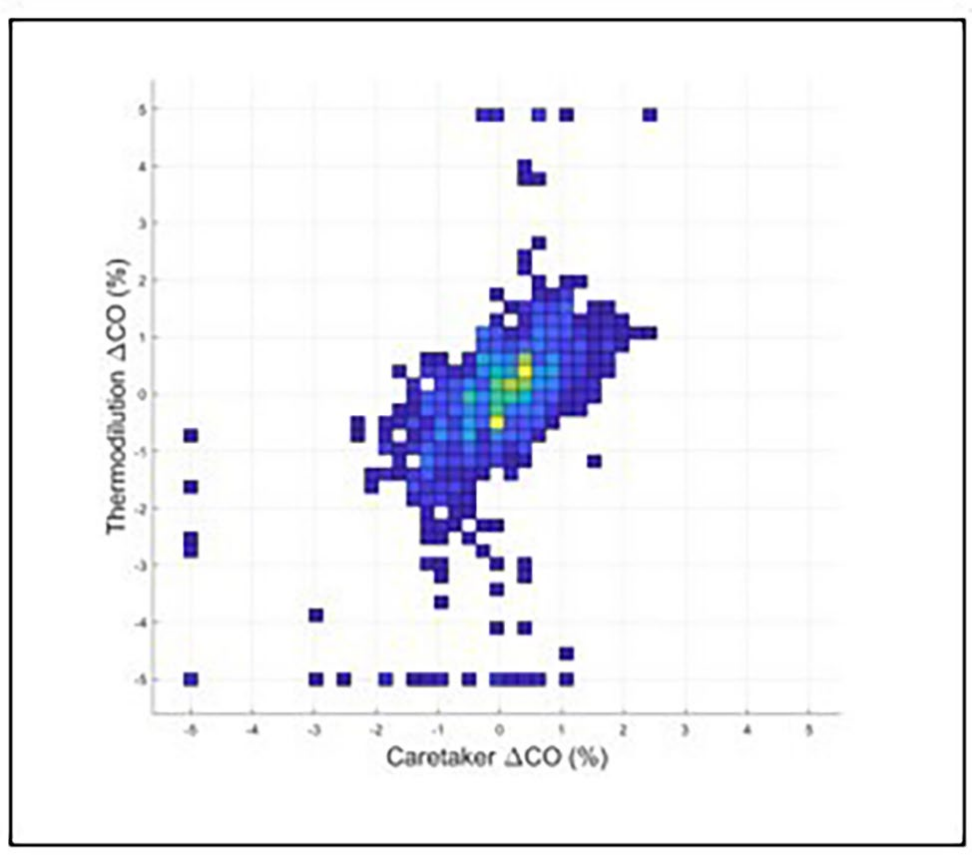
Displays the concordance results (0.69) with zero delay between both data streams. Figure 5 displays the concordance results (0.86) when the optimized delay of 206 s, based on the maximum of the cross-correlation spectrum, is introduced to the Vitalstream data relative to the CCO data

**Fig. 5:**
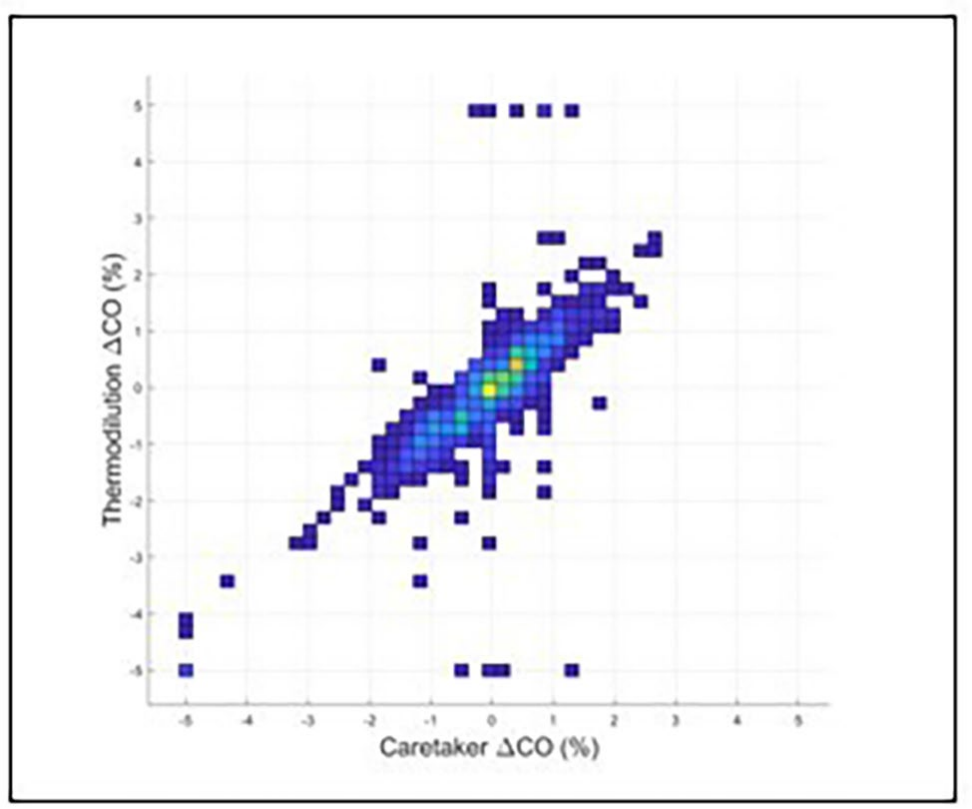
Displays the concordance results (0.86) when the optimized delay of 206 s, based on the maximum of the cross-correlation spectrum, is introduced to the Vitalstream data relative to the CCO data

Figures 4 and 5 display the results of the iterative concordance analysis to establish time delays by calculating the cross-correlation spectrum between the CO data of the Vitalstream and the CO data provided by the CCO system.

## Discussion

The results of this post-process cardiac output comparison study, which compared measurements obtained with the non-invasive VS with TD measurements obtained via TD discrete bolus administration as well as via CCO, are significant in a number of aspects.

First, the results are comparable with those achieved with other non-invasive as well as invasive technologies where cardiac output errors of agreement with TD measurements ranging from approximately 35% to over 50% were reported [1, 2, 9]. The distinction is important because background vibrational noise is highly relevant in the context of cardiac surgeries that are characterized by repositionings, compression of the detection system by the surgical staff, pump startups etc., which affect non-invasive technologies significantly more than pulse analysis approaches utilizing catheter-obtained signals. Even in the context of other non-invasive technologies, the results are remarkable because the VS, based on the PDA approach to pulse analysis, is a passively sensing non-invasive technology, as opposed to actively sensing devices. Those operate based on the principle of Penaz, such as the Clearsight (Edwards Lifesciences) or the CNAP (CN Systems), or applanation tonometery, such as the T-Line systems (Shanshi Medical, Shangqiu, China, formerly Tensys Medical, San Diego). Due to the more aggressive coupling of these active systems to the artery, higher signal to noise ratios would be expected under identical conditions.

Second, the results are significant in the context of the patient population, which is characterized by a wide range of cardiac outputs. Low values were mainly due to ventricular pathologies secondary to heart valve problems. Post-surgery, extremely high cardiac outputs can result from the interplay between better ventricular ejection in the presence of very low systemic vascular resistance (SVR) and the use of inodilators. The incidence of a low-SVR state is common following cardiopulmonary bypass and its incidence varies from 9 to 44% [17].

Another significant result is that an examination of the extremes of CO for this cohort, specifically below 5 l/min and above 8 l/min, did not yield substantially different results from those obtained overall. In contrast, the somewhat unexpected result that the error for calibrated measurements is 2% higher than for uncalibrated measurements is both clinically irrelevant and statistically insignificant in light of the small number of underlying data points.

Finally, the significance of the results of the time delay analysis is that the delay has to be added to the VS data, i.e., its response is significantly faster than that of the CCO system. This is anticipated in light of the well-documented delay time of CCO systems on the order of 5–9 min in response to cardiac output changes [13, 18]. Even in “STAT” mode, in which a faster-response algorithm is used, and which was not used in this study, the response delay to a significant change in cardiac output can be more than 3 min.

A significant consideration is how our results fit into performance expectations. The issue of significant errors between the Gold Standards has been raised by others, who proposed a perhaps more realistic standard of an error of < 45%, giving rise to discussions with the original proponents of the 30% error guideline [19]. In light of those discussions, which seemed to find common ground regarding a more relaxed expectation, we believe that our expectation of < 40% is realistic [20, 21]. As stated previously, results obtained with invasive technologies would appear to support our viewpoint.

While the study was adequately powered, limitations of the study are related to size of the cohort and the number of TD data point comparisons, which were determined by clinical needs. Other limitations are likely associated with a population of relatively sick cardiac patients with a majority having altered hemodynamics and having heart valve disease. While the choice of this population was less than ideal, the use of pulmonary artery catheters and TD has decreased which has limited clinical comparisons between devices in a more diverse population. Finally, it is possible that the improvement in concordance by adding a delay to the VS CO data would have been less clearly resolved if the CCO comparison data set was larger, since a commensurately larger set, and possibly range, of delays would have further broadened the trending response.

## Conclusions

Cardiac output measurements obtained using the VS agreed well with reference discrete and continuous thermodilution cardiac output measurements in cardiac surgery patients, and the faster response time of the VS measurement relative to CCO was demonstrated. The results are significant regarding the goal of expanding access to effective, wireless and readily implemented fluid management monitoring tools to hospital sections previously not covered because of the limitations of traditional technologies. The fast response timeof the VS device provides numerical trend information which is inherently more valuable in managing patient care. Information about trend CO values allows physicians to determine and adjust appropriate patient management plans and apply an integrated approach [22].

This VS device has potential to provide useful hemodynamic information in various settings. Unfortunately, validation studies are usually done in a different clinical setting with a different patient population then the intended target application. Despite these limitations, the agreement between the devices was clinically acceptable with a percent error (PE) of 34.5 to 38% with and without external calibration. The threshold for an acceptable agreement between the VS and TD was considered to be below 40% which is below the threshold recommended by others [20]. Additional research is indicated in a population devoid of significant valvular heart disease and those not receiving vasoactive agents, which was used in all study subjects.

## Data Availability

The dataset used and/or analyzed during this study are available from the corresponding author on reasonable request.

## Abbreviations

VS: Vitalstream
TD: Thermodilution
CO: Cardiac output
CCO: Continuous cardiac output monitoring
PE: Percent error
PDA: Pulse decomposition analysis
SD: Standard deviation
SNF: Single noise factor
OR: Operating room
ICU: Intensive care unit
BP: Blood pressure
LOA: Limits of agreement
CI: Confidence intervals
